# Report of the Committee on Medical Education in Relation to “Demonstrative Midwifery”

**Published:** 1852-01

**Authors:** W. Hooker


### EDITORIAL DEPARTMENT.

*Report of the Committee on Medical Education in relation to "Demonstrative Midwifery."* By W. Hooker, M. D.

This is the title of a pamphlet of eight pages which has lately appeared in this city, heralding the reception of the Transactions of the American Medical Association. The latter work, we are happy to learn, has been issued from the press, and will soon be distributed. The separate publication of Dr. Hooker's report on Demonstrative Midwifery, we presume, is designed especially for this meridian. A large number of copies have been distributed, and, as they are provided for general circulation, they can doubtless be had for the asking by any who are not yet supplied. We are not authorized to say who are the committee having this matter in charge, but we *guess* the readers of this Journal will not be at a loss to know to whom to apply.

Is is now nearly a year since we have had any occasion, as a Journalist, to refer to *Demonstrative Midwifery*—a subject on which, our readers need not be reminded, at a prior date, we felt called upon to express our opinions at some length. The merits of demonstrative teaching in obstetrics we have fully discussed. We are content to rest upon this past discussion, having seen no good reason for a modification of the views then entertained, or for adducing fresh considerations to strengthen our position. We had hoped not again to find it necessary to recur to the subject, thinking that it had already occupied enough of the attention of our readers and ourselves. It is true, that circumstances pertaining to us in our social and professional relations, that have grown out of the free expression of our honest convictions on this subject, are well calculated to provoke additional editorial remarks. We have thus far refrained from yielding to this temptation, partly in consequence of a reluctance that personal matters shall become interwoven with the functions of a journalist, and, in part, because, in this aspect, the subject cannot be divested of pointed personal allusions to other individuals. Our impartial readers will, we believe, unite in saying that our former articles were written, if not with candor, at least, with a due regard to courtesy. Such, at all events, we aver to have been our aim; and we are certain that, in this point of view, they will be likely to be appreciated, should we ever deem it incumbent to undertake a faithful and complete history of the midwifery crusade, not restricting ourselves, as hitherto, to a mere discussion of the abstract merits of the subject. Justice to ourselves may require such a history, but we do not propose now to enter upon it.

The Report of the Chairman of the Committee on Medical Education, on Demonstrative Midwifery, being before us, and purporting to be extracted from the Transactions of the American Medical Association, it claims, at our hands, some notice. The subject is introduced in the following words—we quote the first three paragraphs of the Report:

"At the last meeting of the Association, there was referred to the consideration of this Committee a subject which has recently excited much interest throughout the profession in this country, and upon which there has been much difference of opinion. We allude to what has been termed Demonstrative Midwifery.

"Though the Committee think that the examination of this subject could have been made thoroughly and satisfactorily in the journals of the profession, and that its reference to us was, therefore, unnecessary; and though we have felt, from the first, that, if referred at all, it should have been referred to the Committee on Obstetrics; yet, in obedience to the direction of the Association, we have prepared our views in regard to it, and are ready to present them. We ask, however, that it may be done as a special report by itself. The main report has, in its view of the general subject of medical education, a unity and completeness which would be impaired by the introduction of matter that is incongruous and irrelevant to its scope and design.

"As the reference of this subject to the Committee grew out of the course which had recently been pursued in one of our medical schools in teaching midwifery, it will be proper to present the facts of the case before we proceed to state the rules by which we think that the practice of teachers in this branch of medicine should be governed."

He then goes on to state facts respecting the mode of instruction in midwifery pursued at Buffalo.

Dr. Hooker appears to have strangely misapprehended the instructions of the Association to the committee of which he was chairman. This will be apparent on comparing the above quotation, setting forth the scope of his report, with the resolution adopted by the Association committing the subject to the Committee on Medical Education. The resolution, with the preamble, reads thus:—"Whereas clinical instruction in medicine and surgery is now generally acknowledged to be essential to the proper qualification of students for the practice of these branches of our profession; *and whereas, it must be admitted that clinical instruction in midwifery would be equally valuable,* therefore,

"*Resolved,* that the Committee on Medical Education *be instructed to inquire whether any practicable scheme can be devised to render instruction in midwifery more practical than it has hitherto been in the medical schools of the United States,* and report at the next meetiug of the Association."*

This resolution was offered by Prof. Miller, of Ky.; it was offered under a suspension of the rules, and *adopted unanimously.* Here we may be permitted to say, that from no other member of the Association could such a resolution more appropriately come than from Prof. Miller. Prof. M. is not only a distinguished teacher of midwifery, of large experience, in one of the most flourishing of the medical institutions of this country, but he is known, both at home and abroad, as the author of one of the most original and profound of the works embraced in the medical literature of the present time. Such a man would be likely to understand, fully and clearly, the true defects in the present system of obstetrical instruction, and to appreciate just what is wanting to remedy these defects.

The preamble, it will be observed, states plainly that clinical instruction in midwifery is a desideratum. The resolution is based on this statement, and the Committee on Medical Education are instructed to inquire "whether any practicable scheme cannot be devised to render instruction in midwifery more practical than it has been hitherto been in the medical schools of the United States." This resolution passed unanimously. The Association, therefore, affirmed, as strongly as possible, the fact that clinical instruction in midwifery was to be desired, and that some practicable scheme should be devised to secure this desirable end.

Now, we repeat, Dr. Hooker appears strangely to have overlooked the plain meaning of the resolution referred to the committee of which he was chairman. He says that he is called upon to report on the subject of Demonstrative Midwifery; he goes on to give an account of the particular demonstration at Buffalo, in 1849-50, and considers, evidently, the term *demonstrative* to refer exclusively to an *ocular* exhibition of the process of parturition. The entire Report has relation to this ocular exhibition exclusively. He considers nothing else, save the use of the speculum, which is wholly extrinsic to the matter of clinical instruction in midwifery.

---

* The italics are ours.

The *instructions* contained in the resolution, while they did not of course preclude the utterance of the opinions the author of the Report has expressed, called for much more. They rendered it incumbent on the committee to inquire whether some practicable scheme could be devised to render clinical instruction in midwifery more practical. The phrase "demonstrative midwifery" (a phrase which some persons affect not to comprehend,) was not mentioned in the resolutions. No allusion was made to the fact (a fact to which we shall ever refer with satisfaction,) that the medical school at Buffalo had taken the lead in the matter, and had made a successful effort to render instruction in midwifery more practical. In short, the object of the resolution has not been met, and for all useful purposes, it remains to be referred to a future committee. Another point seems, singularly enough, not only to have been overlooked by Dr. Hooker, but to be lost sight of by many who have become interested in this subject. It appears to be considered that all the advantage claimed in behalf of "demonstrative midwifery," as pursued at the medical school of Buffalo, relates to the *ocular* exhibition. The *demonstration* is supposed to embrace this exhibition alone. This, however, is but a part, and perhaps a minor part of the plan of demonstrative teaching. We desire not to be misunderstood, for a moment, in this remark. We regard the ocular exhibition as important, because highly useful, and entirely proper, inasmuch as it conduces to instruction. Our views on this point are in no wise changed. But the demonstration at the Buffalo Medical College involved much more than the ocular exhibition of the conclusion of the parturition. The stethoscopic signs of pregnancy; the mode of examination by the touch, and the information therefrom derived in the different stages of labor; the professional deportment of the practitioner in the parturient chamber; the details of the management of labor; the putting to bed, and all the offices of the accoucheur, both to mother and child, were witnessed and impressed by *demonstration.* We shall not stop, now, to ask whether all this can be done as well by plates and buckskin manikins; we have considered that inquiry heretofore. The ocular exhibition (in which it was not proved that the genitalia could be seen,) lasted but five minutes. The parties interested in the advocacy of demonstrative midwifery can well afford to dispense with that useful element of this mode of instruction, and still have sufficient left to refer to as a praiseworthy effort "to render instruction in midwifery more practical than it has hitherto been in the medical schools of the United States."

To return to the report — Dr. Hooker repudiates the idea, which he states to be as prevalent as false, that a female sacrifices modesty by submitting to any exposure which is necessary either in the practice of medicine or the instruction of students. A different opinion, coming from a physician of any intelligence, can only spring from some malicious intent. Dr. H. also alludes to the absurdity of attaching any indelicacy to the use of the sense of sight, in distinction from the sense of touch. He then proceeds to state the grounds on which the ocular exhibition of the process of parturition is advocated. We quote his remarks under this head in full:

"There are four advantages claimed to attend an exposure to the sight of the conclusion of the process of labor. We will notice each of these separately:—

1st. The student sees the manner in which the head of the child, or whatever part presents, emerges from the os externum. All that is of practical use in regard to this can be so well learned from description and plates, and from exhibitions on the machines commonly used in the lecture room, that there is clearly no need of an exhibition of the living subject to prepare the student on this point for practice. And whatever he does not learn in regard to it by these means, can be learned by the touch in the first case upon which he is called to attend. No practitioner ever had any desire to see the presenting part emerge under the arch of the pubis for any additional knowledge that might be gained by such an exposure.

2d. By an exposure of the parts to the sight at the conclusion of the labor, the student is impressed with the importance of supporting the perineum. On this point we simply remark, that, if any student cannot be properly impressed with the importance of this act by the teachings of his preceptor without ocular demonstration, he has too dull an appreciation of truth and responsibility to take upon himself the office of a physician.

3d. The exposure contended for shows the student the manner in which the perineum should be supported. In learning how to do a manual operation, which, when learned, is to be done without the guidance of sight, the use of sight is not needed except when the operation is a difficult or complicated one. That supporting the perineum is an operation of this character, cannot be pretended. It is about as simple an act as can be conceived of, and the student who needs the aid of his eyes to learn how to do it had better retire at once to some operation that requires less tact and talent than the practice of medicine. In the case before us, the accoucheur used a napkin, which, unless it was very adroitly managed, must have prevented the twenty spectators from seeing very distinctly the exact manner in which he supported the perineum.

4th. This exposure verified, to the satisfaction of the students, the diagnosis of the professor in regard to the position of the child. But a resort to such evidence for this purpose is certainly unnecessary. The diagnosis can be verified by the touch on the part of the student during the progress of the professor, which ought to be satisfactory.

Granting all that can be claimed with any plausibility for the advantages mentioned, they are not of sufficient value to make it proper that a woman, in the hour of her extremity, should be made the subject of a public exhibition."

We shall not comment on the above quotation. We have considered these points in our former articles, and we are satisfied to ask the reader to refer to them in connection with Dr. Hooker's remarks. We recognize, of course, the full right of Dr. H., or any one, to differ from us in opinion, claiming the privilege of adhering to our own until more solid arguments are adduced for their disproval than are contained in this Report. We know Dr. H. well enough to be well assured that to say this will not be considered disrespectful to him. Honest differences of opinion among gentlemen do not involve breaches of courtesy nor impair friendly regard. On this point, Dr. H. speaks as every honorable man must think. He says:

"It is wholly a professional question, and should be discussed by the profession in a calm, considerate, and dignified manner. It is no subject for newspaper warfare, nor for a warfare in medical journals in newspaper style. No aspersions should be cast upon the motives of the parties interested. A distinction should always be made between those who honestly differ in opinion from their brethren, and those who are governed by the base motives of the mercenary charlatan; and also between those who oppose practices which they think to be improper, with sincere, though perhaps too ardent zeal, and those who do this from a malicious or meddling spirit."

The Report continues—"But we not only object to the mode of instruction adopted in the plan at Buffalo as unnecessary, but we object to it, also, as being utterly *incompetent to give the student that knowledge which he needs in the practice of obstetrics.* It cannot take the place at all of what may properly be termed Clinical Instruction in Midwifery." Now, it was never contended, or claimed for a moment, that the ocular part of the demonstration was to take the place of more extended clinical instruction in midwifery. This must be evident from what has been already said in this article. The *exposure* was but an element of a system of clinical instruction embracing all that is calculated to render such instruction full, complete, and practical.

In conclusion, we observe printed in a conspicuous place in the pamphlet gotten up for gratuitous distribution in this vicinity, a resolution expressing the unanimous approval by the American Medical Association of the opinions contained in the Report of the Committee on Medical Education in respect to demonstrative midwifery. They who have been at the pains and expense of procuring a pamphlet edition of the Report to be circulated in this community, show their desire that the action of the Association in this matter should be known. We have no reluctance to contribute our assistance to diffuse information on this point. We beg, therefore, all our readers clearly to understand that, while the American Medical Association of 1850 unanimously affirmed clinical instruction in midwifery to be just as essential as clinical instruction in medicine and surgery, the American Association of

1851 declare, unanimously, that the *ocular* demonstration of parturition is not necessary. We know that certain members of the Association present at the meeting of 1851, were ardent advocates of ocular demonstration. They, doubtless, felt no disposition to obstruct the current tending to the above vote of the Association, nor have we any disposition to take exceptions to such action. Opinions formed by those who are accustomed to think for themselves will not be likely to be affected by the tenor of a resolution adopted without discussion, by a public body, a large portion of the members of which had perhaps given to the subject little or no consideration. It may be imagined that the moral force of the action of the Association can be brought to bear on popular prejudice so as to damage the private interests of individuals. With respect to giving the Report of Dr. Hooker such a direction, all we will say at this time is, that, were the effort successful, the able author of "Physician and Patient" would be one of the last persons to feel complimented by being made an involuntary instrument in schemes of personal animosity.

